# Impact on Health-Related Quality of Life after Different Aerobic Exercise Programs in Physically Inactive Adults with Overweight/Obesity and Primary Hypertension: Data from the EXERDIET-HTA Study

**DOI:** 10.3390/ijerph17249349

**Published:** 2020-12-14

**Authors:** Mikel Tous-Espelosín, Ilargi Gorostegi-Anduaga, Pablo Corres, Aitor MartinezAguirre-Betolaza, Sara Maldonado-Martín

**Affiliations:** 1 GIzartea, Kirola eta Ariketa Fisikoa Ikerkuntza Taldea (GIKAFIT) Society, Sports, and Physical Exercise Research Group, Department of Physical Education and Sport, Faculty of Education and Sport-Physical Activity and Sport Sciences Section, University of the Basque Country (UPV/EHU), 01007 Vitoria-Gasteiz, Spain; mikel.tous@ehu.eus (M.T.-E.); ilargi.gorostegi@ehu.eus (I.G.-A.); pablo.corres@ehu.eus (P.C.); aitor.martinezdeaguirre@ehu.eus (A.M.-B.); 2 Bioaraba Health Research Institute, 01009 Vitoria-Gasteiz, Spain

**Keywords:** SF-36 questionnaire, supervised exercise, physical health, mental health, high-intensity interval training, low-volume training

## Abstract

Primary hypertension (HTN) and obesity are associated with a worse health-related quality of life (QoL). This research was carried out to analyze the health-related QoL measurements in a physically inactive and obese population with HTN (n = 253) in comparison to a HEALTHY sample (n = 30), to determine the HTN sample changes in QoL following different (high-volume moderate-intensity continuous training, high-volume high-intensity interval training (HIIT), low-volume HIIT) 16-week supervised aerobic exercise training (ExT) programs compared to attention control, and to assess the differences in QoL variables between the different ExT programs. The SF-36 questionnaire was used to assess health-related QoL. At baseline, HTN showed lower scores (*p* < 0.05) in physical function (88.6 vs. 99.2), general health (63.3 vs. 82.4), vitality (58.2 vs. 68.7), social functioning (88.5 vs. 95.2), and mental health (76.1 vs. 81.8) compared to HEALTHY. Following intervention, all HTN subgroups showed higher (*p* < 0.05) vitality, but physical functioning and general health significantly improved only in the ExT groups, with even better values in general health for both HIIT subgroups. Only the low-volume HIIT showed positive changes (*p* < 0.05) in social functioning (∆ = 6.9%) and mental health (∆ = 6.4%) domains after the intervention. These results highlight the important role of supervised exercise in improving physical and psychological health.

## 1. Introduction

Primary hypertension (HTN), with an overall prevalence in adults around 30–45%, is recognized as a major risk factor for cardiovascular disease; chronic kidney disease; peripheral artery disease; and cognitive decline disease [1]. A previous systematic review and observational studies concluded that the quality of life (QoL) of individuals with HTN is worse than that of normotensive ones, with better values in the physically active population compared to sedentary ones [2,3,4]. Therefore, a non-pharmacological treatment of HTN is recommended, involving lifestyle interventions that might result in lower blood pressure (BP) and avoid the need for medication to improve the health status of this population [1,5].

Exercise training (ExT) contributes to the management of HTN, with health promotion, greater cardiorespiratory fitness, decreasing mortality and cardiovascular morbidity, and reduced BP values [6]. Experimental studies indicate that high-intensity interval training (HIIT) produces superior benefits than moderate-intensity continuous training (MICT) in cardiorespiratory fitness improvement, but similar enhanced control of BP [6,7]. Adding to that, the low-volume HIIT (i.e., ≤10 min of high-intensity effort in one exercise session) has emerged as a time-efficient and effective training method for improving health [7].

It is well known that regular physical activity improves health-related QoL contributing to perceived well-being [8], with greater improvements in interventions supervised by exercise specialists [9]. In a recent study, comparing the effects of HIIT and MICT in patients with fibromyalgia, both exercise groups showed significant and similar improvements for pain, functional capacity and QoL compared to the control group [10].

Many questionnaires have been developed to assess population health, but finding a generic questionnaire that is easy to administer, acceptable, valid, and short has been a difficult task. One of the most used questionnaires has been the Nottingham health profile due its acceptability and shortness [11]. However, it has been criticized for its inability to detect low levels of disability that are important not only clinically but also for respondents [12]. The 36-item short-form health survey questionnaire (SF-36) is not just a potentially valuable tool for medical research, it has also served to assess the QoL in different populations with diseases, such as fibromyalgia, knee osteoarthritis, and chronic low back pain [13], cancer [14], psoriasis [15], and chronic kidney disease [16]. On the other hand, in individuals with HTN, while the beneficial effects of MICT [17] and a walking program [18] on QoL assessed with the SF-36 have been confirmed, the relationship in response to HIIT programs, differing in volume, has not been examined in this population. 

Therefore, the main purposes of this study were: (1) to compare the health-related QoL in physically inactive individuals with obesity/overweight and HTN with that of HEALTHY ones; (2) to determine changes in QoL following different (high-volume MICT, high-volume HIIT, low-volume HIIT) 16-week supervised aerobic ExT programs compared to attention control (AC) in inactive individuals with obesity/overweight and HTN; and (3) to analyze the possible differences in QoL variables between the different ExT programs.

## 2. Methods

### 2.1. Study Design

Data from the present study were taken from the EXERDIET-HTA research including two sub-studies: (1) a cross-sectional analyzing the health-related QoL measurements in physically inactive and obese population with HTN in comparison to a HEALTHY sample, and (2) a multi-arm parallel, randomized, single-blind controlled experimental trial comparing the effects of different 16-week aerobic ExT programs (performed 2 days/week) combined with dietary intervention in physically inactive, overweight/obese individuals with HTN (www.clinicaltrials.gov, number NCT02283047). The study protocol was approved by the ethics committee of the University of the Basque Country (UPV/EHU, CEISH/279/2014) and clinical investigation of Araba University Hospital (2015-030), and all participants provided written informed consent before any data collection. The medical staff was blinded to the participant randomization process. Examinations at baseline and follow-up were performed in the same laboratory setting and by the same researchers. The design, selection criteria, and procedures for the EXERDIET-HTA study have been detailed previously [19].

### 2.2. Participants

**Figure 1:**
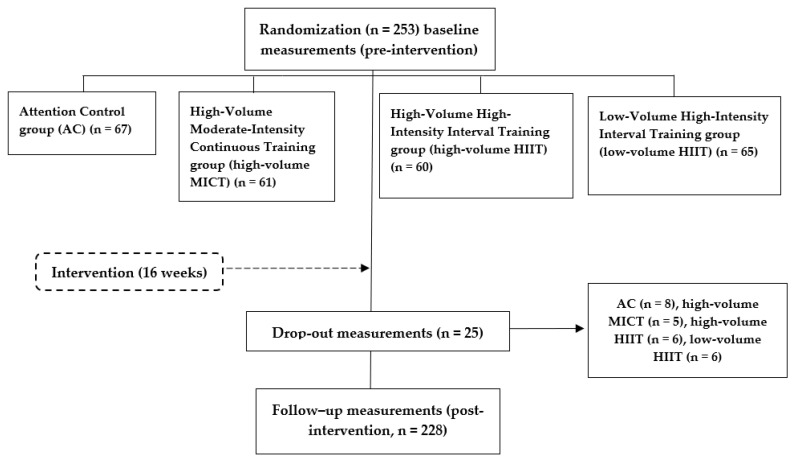
Flow diagram of the EXERDIET-HTA study from recruitment to the follow-up measurements post intervention.

A flow chart of the participants in this study is shown in Figure 1. Participants were recruited from the medical services (cardiology) and local media (university media, newspapers, radio, and television). Interested individuals were invited to contact the research team. A sample of 253 individuals aged between 24 and 69 years (53.7 ± 7.9 years) took part in the study, 161 men and 92 women. All participants were physically inactive (i.e., they were below the Global Recommendations of Physical Activity for health set by the World Health Organization) [20], overweight/obese (i.e., body mass index ≥25 kg/m^2^ or 30 kg/m^2^, respectively), and had the diagnosis of HTN (i.e., mean systolic BP ≥140 mmHg and/or diastolic BP ≥90 mmHg or under antihypertensive pharmacological treatment) [21]. Participants with no diagnosis of HTN were assessed with ambulatory BP monitoring (ABPM) to confirm the HTN status by a cardiologist. All other inclusion and exclusion criteria were specified in the study protocol [19]. Data were retained from those participants who had measurements in anthropometry, ABPM, and cardiorespiratory fitness using the metabolic gas analysis system.

In addition to the EXERDIET-HTA study participants, another group was created to allow for comparison to a healthy control population (HEALTHY, n = 30, 40.0 ± 9.0 years), who did not receive any type of intervention. Only baseline measurements were assessed for comparison to the EXERDIET-HTA study participants. HEALTHY participants were recruited from the community through university media. Inclusion for HEALTHY criteria was to be 25–55 years of age and exclusion criteria were being pregnant, currently breastfeeding, taking regular medication, or having any known medical conditions, had abnormal findings on physical examination (including blood pressure ≥140/90 mmHg, or overweight ≥25 kg/m^2^), or had abnormal results on screening test (rest and exercise electrocardiogram).

### 2.3. Measurements

A full description of the study protocol was previously presented elsewhere [19]. A brief explanation of the main measures is presented below.

Anthropometry measurements for the assessment of body composition included stature (SECA 213, Hamburg, Germany), total body mass, and body mass index (BMI) (Tanita, BF 350, Arlington Heights, IL, USA), and waist circumferences (SECA 200, Hamburg, Germany).

Ambulatory BP monitoring was measured with an oscillometric ABPM (6100 and 7100 recorders, Welch Allyn, New York, NY, USA). The device measured BP an entire day, at 30-min intervals during the daytime, and at 60-min intervals during nighttime. The variables registered from the ABPM were mean values of systolic BP and diastolic BP during the day and night periods.

Physical fitness was determined by performing a symptom-limited cardiopulmonary exercise test on an electronically braked Lode Excalibur Sport cycle ergometer (Groningen, The Netherlands). The test started at 40 W with a gradual increment of 10 W every minute applied until volitional exhaustion. The expired gas was analyzed using a commercially available metabolic cart (Ergo Card, Medi-soft S.S, Belgium Ref. USM001 V1.0). Achievement of peak oxygen uptake criteria has previously been defined [22] and was assumed with the presence of two or more of the following criteria: volitional fatigue (>18 on Borg Scale), peak respiratory exchange ratio ≥1.1, achieving >85% of age-predicted maximum heart rate, and failure of oxygen uptake and/or heart rate to increase with further increases in work rate. Absolute and relative indications for terminating the exercise test were taken into account [23].

The health-related QoL was assessed using the Spanish version of the SF-36 questionnaire [24]. The items of the questionnaire report both positive and negative states of “physical component summary” and “mental component summary”, identifying eight dimensions of health: physical functioning, role-physical, bodily pain, general health, vitality, social functioning, role-emotional, and mental health. For each dimension of the SF-36, the items are coded, added, and transformed into a scale with a path from 0 to 100 (higher scores indicating higher levels of health-related QoL) using the algorithms and indications that the scoring and interpretation manual of the questionnaire offers [25]. Psychometric evaluation of instruments targeting two independent constructs of physical and mental health has been recently published and validated [26]. Once the SF-36 questionnaire was explained, each participant answered it on their own.

### 2.4. Intervention

After baseline measurements, EXERDIET-HTA participants were randomly allocated into one of the four intervention subgroups stratified by sex, systolic BP, BMI, and age using a time-blocked computerized randomization program. The medical staff was blinded to participant randomization assignment. The four intervention subgroups were: three supervised ExT groups (i.e., high-volume MICT, high-volume HIIT, low-volume HIIT) on two non-consecutive days per week under supervision by exercise specialists for 16 weeks; and one attention AC, with only physical activity advice participating in at least 30 min of moderate-intensity aerobic exercise (walking, jogging, cycling, or swimming) for 5–7 days per week blended with some dynamic resistance exercises [19]. The high-volume MICT group performed 45 min of aerobic continuous exercise at moderate intensity, whereas the high- and low-volume HIIT groups performed 45 and 20 min, respectively. In the HIIT groups, they alternated high- and moderate-intensities performing protocols previously published [19]. All participants underwent a hypocaloric DASH diet. The diet was designed to provide 25% less energy than their daily energy expenditure and to achieve a weekly loss in body mass of between 0.5 and 1.0 kg in accordance with the recommendations of the American Diabetes Association and the Spanish Society for the Study of Obesity [27]. The diet contained approximately 30% fat, 15% protein, and 55% carbohydrates and was designed following the DASH diet. To ensure participant compliance with the DASH diet every two weeks participants were weighed and received encouragement and advice alongside nutritional counseling to aid compliance [7].

### 2.5. Statistical Analysis

The values of each variable were obtained considering the mean and standard deviation (SD). Data were considered for the entire sample and presented in groups. Analysis of variance (ANOVA) was used to determine if there were significant pre-intervention differences between groups. In contrast, the differences between pre-intervention (T_0_) and post-intervention (T_1_) were analyzed using a Student-t test related to the sample in each variable. Analysis of covariance (ANCOVA) was used to examine the delta (∆) score for each group (AC, high-volume MICT, high-volume HIIT, low-volume HIIT) and Bonferroni correction was used to determine the level of significance when a significant main effect was found. Furthermore, the differences between AC vs. ExT were analyzed using the Helmert contrast. Data were analyzed according to the intention-to-treat principle. Statistical significance was set at *p* < 0.05. The statistical analyses were performed with the SPSS version 25.0 software package. The power calculation was completed using the G*Power 3 analysis program [28]. The required sample size was determined for the primary outcome variable (systolic BP) of the EXERDIET-HTA study. It was identified that adequate power (0.80) to evaluate differences in the current design consisting of four experimental groups would be achieved with 164 people (41 each group, α = 0.05, effect size f = 0.27) based on the pilot study with an SD of 9 mmHg.

## 3. Results

At baseline, answering the first goal of the study (a cross-sectional analysis), significant differences were presented in participants’ characteristics between the two samples (i.e., HEALTHY vs. EXERDIET-HTA) (Table 1). Thus, whereas HEALTHY were classified as normal body composition and BP values, as well as good cardiorespiratory fitness, EXERDIET-HTA individuals showed obesity values in addition to low cardiorespiratory fitness. These results have been previously presented and further explained [29]. In contrast, analyzing the domains of health-related QoL, EXERDIET-HTA participants showed lower scores in physical function (88.6 vs. 99.2, *p* < 0.001), general health (63.3 vs. 82.4, *p* < 0.001), vitality (58.2 vs. 68.7, *p* < 0.001), social functioning (88.5 vs. 95.2, *p* = 0.002), and mental health (76.1 vs. 81.8, *p* = 0.001) in comparison with the HEALTHY population (Table 1). Adding to that, the physical and mental component summaries were also lower (50.3 vs. 54.9, *p* < 0.001, 50.9 vs. 53.1, *p* = 0.036, respectively) in the EXERDIET-HTA compared to the HEALTHY group. Further, there were no significant differences between EXERDIET-HTA subgroups (*p* > 0.05).

Following 16 weeks of intervention, answering the second and third goals of the study (controlled experimental trial), positive changes (T_0_ vs. T_1_, *p* < 0.05) were observed in the total EXERDIET-HTA sample in some of the domains of the SF-36 questionnaire (Table 2), such as physical functioning (difference %, ∆ = 4.9%), general health (∆ = 10.8%), vitality (∆ = 12%), social functioning (∆ = 4.4%), and mental health (∆ = 3.5%). Vitality was the unique domain improving with higher (*p* < 0.05) values in each of the four groups. Further, physical functioning (*p* = 0.011), and general health (*p* = 0.001) differed when AC vs. ExT subgroups were compared, with higher values in ExT groups (“Physical functioning”: high-volume MICT, mean difference 4.189, 95% confidence interval (CI) −1.184–9.563; high-volume HIIT, mean difference 5.169, 95% CI −0.109–10.448; low-volume HIIT, mean difference 3.007, 95% CI −2.366–8.382. “General health”: high-volume MICT, mean difference 5.319, 95% CI −2.531–13.171; high-volume HIIT, mean difference 9.237, 95% CI 1.525–16.949; low-volume HIIT, mean difference 9.829, 95% CI 1.977–17.680). Further, a favorable effect of the intervention was also observed in “general health” in high-volume HIIT (*p* < 0.001, mean difference -10.135, 95% CI −14.801 + 5.470), and low-volume HIIT (*p* < 0.001, mean difference −6.218, 95% CI −10.261 + 2.174) vs. AC, with higher values in both ExT subgroups. Likewise, the “vitality” domain in low-volume HIIT showed higher (*p* = 0.043) values than AC. Interestingly, after intervention positive differences (*p* < 0.05) in “social functioning” (∆ = 6.9%) and “mental health” (∆ = 6.4%) domains were only found in the low-volume HIIT subgroup, leading consequently to higher (∆ = 6.3%, *p* < 0.05) “mental component summary” (Table 2).

## 4. Discussion

This research was carried out to analyze the health-related QoL measures in a physically inactive and obese population with HTN in comparison to a HEALTHY sample, and to determine the effects, after a 16-week ExT intervention period, on the hypertensive and physically inactive sample with obesity both as a whole and also on each of the subgroups of the study. The main findings of the present study were: (1) the HEALTHY group showed higher scores in physical function, general health, vitality, social functioning, and mental health in comparison with the HTN participants; (2) after a 16-week intervention period “vitality” showed higher values in all HTN groups; (3) “physical functioning” and “general health” scores improved more in the supervised ExT groups compared with the AC, with better values in general health in both HIIT groups; and (4) only the low-volume HIIT subgroup showed positive changes in “social functioning” and “mental health” domains after the intervention.

As one might expect, the scores of health-related QoL in HEALTHY individuals were higher (better) than those of participants with HTN, obesity, and physical inactivity. These results coincide with those by previous studies with a significant impact of chronic conditions [30] and HTN [3] on physical and mental health, and consequently the added burden associated with comorbidity. Thus, according to the SF-36 Health Survey [25], EXERDIET-HTA participants were represented in the low-medium range (i.e., 25–50th percentile) related to general health, vitality, and mental health, with a general medium-high perception of physical component summary (50–75th percentile), and low-medium perception of mental component summary (25–50th percentile).

European guidelines for the management of arterial HTN [1] claim that healthy lifestyle choices, including a healthy diet and physical activity, can prevent or delay the onset of HTN and reduce cardiovascular risk. In the present study, the 16-week intervention program showed the benefits of health-related QoL in some domains, such as “vitality” in all groups, reinforcing the power of a healthy lifestyle to make you feel strong, active, and energized. Therefore, the potential mechanism of physical activity for improving cognitive processes, antidepressant effects, and even inducing a sense of well-being has been previously discussed [31]. At a physiological level, acute exercise appears to improve mood by activating specific cortical areas and inducing the release of neurotransmitters (i.e., serotonin, dopamine, or norepinephrine) and trophic factors that contribute to adherence to a regular physical activity program by increasing the feeling of well-being and also inhibiting the nerve fibers that transmit pain [32].

Moreover, the supervised ExT groups did demonstrate greater improvements in the physical area (physical functioning and general health) compared to AC. These results are concomitant with the improvements in body composition, cardiorespiratory fitness, BP, and cardiovascular risk previously published in the studied sample [7,33]. This is the effect of multiple physiological adaptations that translate into a better physical functioning with fewer limitations in performing all types of physical activities and increase personal evaluation in general health [25]. It is well recognized that good cardiorespiratory fitness has become a key vital sign, accepting the role of exercise in cardiovascular medicine and the molecular mechanisms underlying the benefits of exercise [34]. Further, a growing body of evidence suggests that exercise involving HIIT induces larger benefits and is more effective for improving cardiovascular and metabolic health compared to MICT [35]. In the present study, this favorable effect is also presented in the “general health” item, when both HIIT groups showed higher positive values compared to the AC group, showing the safety and efficacy of this exercise design also in physically inactive people with obesity and HTN. Likewise, it was important to observe that supervised ExT programs by exercise specialists, as in previous studies [9,18,36] had better results compared to only physical activity advice reporting a positive relationship with health-related QoL. These results reinforce the general guidelines and put aside the idea of “one size fits all” recommending an exercise program designed in a systematic and individualized manner in terms of the frequency, intensity, time, and type (i.e., FITT principle) [37]. Additionally, it has also been observed that group interventions favor the development of bonds among individuals through the exchange of experiences and feelings during these activities, improving the individual’s well-being and mental health [38]. Likewise, participation in regular group classes seems to lead to a significant decrease in perceived stress and an increase in physical, mental, and emotional QoL [39].

After analyzing the health-related mental domain, the low-volume HIIT program was the only group improving the “social functioning” (↑6.9%) and “mental health” (↑6.4%) items at follow-up. Recent investigation has shown very good results in individuals with severe obesity after performing a low-volume HIIT program for 12 weeks inducing significant improvements in QoL [40]. Thus, low-volume HIIT programs are emerging as a high level of acceptance, as long as feasible, time-efficient, effective, and enjoyable exercise programs in overweight/obese and inactive populations [41]. The aforementioned results underpin the final improvement of the mental component summary in the present study as a demonstration of the efficacy of low-volume HIIT.

The current study has several strengths, including a relatively large (n = 253), carefully screened, well-characterized group of non-physically active adults with obesity and HTN. There are, however, some limitations of this study that should be considered. First, the current study had only 36.4% of women, which does not represent a division by equal sex. In addition, the perspective between the sexes could be different when highlighting the questionnaire, since women usually present lower values in all multi-items compared to men [25]. As this raises statistical problems, future studies, particularly those using interventions and questionnaires, should seek to recruit equal numbers. Second, as the SF-36 is a subjective questionnaire, it is difficult to assess all its results accurately. Furthermore, future studies with a larger sample should seek to determine whether the different training programs have greater benefits in the different sections.

## 5. Conclusions

Physically inactive adults with obesity and HTN showed a worse health-related QoL profile compared to a HEALTHY population. A 16-week supervised aerobic ExT and nutritional intervention program for hypertensive individuals with obesity and physically inactive was effective in increasing vitality, with better scores in the physical area in the supervised ExT groups compared to AC. Low-volume HIIT may induce more relevant positive effects on social functioning and mental health compared to high-volume programs. These results highlight the important role of supervised exercise in improving physical and psychological health.

## Figures and Tables

**Table 1:** Characteristics of the studied population and scores for eight variables of SF36 by group and subgroups at baseline.

				EXERDIET-HTA SUBGROUPS				
Variables	HEALTHY (n = 30)	EXERDIET-HTA (n = 253)	*p _HEALTHY_*_vs. *EXERDIET-HTA*_	AC (n = 67)	High-Volume MICT (n = 61)	High-Volume HIIT (n = 60)	Low-Volume HIIT (n = 65)	*p* Value Subgroups
Sex (men/women)	12/18	161/92	<0.001	41/26	38/23	39/21	43/22	0.934
Age (yrs)	40.0 ± 9.0	53.7 ± 7.9	<0.001	52.7 ± 8.4	54.2 ± 7.2	53.0 ± 8.6	54.7 ± 7.2	0.339
Body mass (kg)	66.1 ± 10.5	91.5 ± 15.2	<0.001	91.3 ± 15.2	92.5 ± 16.8	90.1 ± 15.1	91.3 ± 14.0	0.826
BMI (kg/m^2^)	23.1 ± 2.6	32.1 ± 4.2	<0.001	32.3 ± 4.4	32.3 ± 4.4	31.7 ± 3.7	32.0 ± 4.3	0.799
Waist (cm)	74.7 ± 8.1	103.2 ± 11.2	<0.001	103.3 ± 11.1	104.1 ± 12.8	101.8± 11.0	103.1 ± 10,1	0.667
SBP (mmHg)	114.0 ± 6.6	135.8 ± 12.1	<0.001	138.2 ± 13.4	133.9 ± 11.1	134.3 ± 10.0	136.5 ± 13.2	0.167
DBP (mmHg)	68.1 ± 7.2	78.0 ± 8.1	<0.001	79.0 ± 8.3	75.8 ± 8.1	78.9 ± 7.6	78.5 ± 8.5	0.077
V̇O_2peak_ (mL·kg^−1^·min^−1^)	48.1 ± 8.1	22.6 ± 5.5	<0.001	23.3 ± 6.3	21.8 ± 5.2	22.7 ± 4.9	22.6 ± 5.5	0.451
Physical Functioning	99.2 ± 2.3	88.6 ± 12.2	<0.001	89.2 ± 11.8	88.6 ± 12.5	89.3 ± 11.2	87.2 ± 13.3	0.757
Role-Physical	96.0 ± 18.4	90.8 ± 25.0	0.165	92.5 ± 21.8	86.5 ± 28.7	94.6 ± 21.6	89.6 ± 27.2	0.228
Bodily Pain	81.3 ± 11.2	78.2 ± 19.7	0.189	75.3 ± 19.2	78.5 ± 18.7	80.9 ± 18.5	78.4 ± 22.3	0.482
General Health	82.4 ± 13.0	63.3 ± 16.6	<0.001	65.0 ± 18.1	63.9 ± 15.2	62.4 ± 15.1	62.1 ± 17.9	0.735
Vitality	68.7 ± 9.9	58.2 ± 15.0	<0.001	58.1 ± 15.6	57.6 ± 14.8	60.1 ± 14.4	56.9± 15.2	0.704
Social Functioning	95.2 ± 10.0	88.5 ± 17.2	0.002	88.8 ± 17.3	87.9 ± 16.8	89.0 ± 17.1	88.3 ± 17.8	0.991
Role-Emotional	91.4 ± 21.0	89.5 ± 27.4	0.704	92.0 ± 24.7	85.8 ± 30.7	91.7 ± 24.3	88.2 ± 29.7	0.576
Mental Health	81.8 ± 7.3	76.1 ± 14.2	<0.001	75.0 ± 14.1	78.2 ± 12.9	76.1 ± 15	75.5 ± 14.7	0.592
Physical Component Summary	54.9 ± 4.2	50.3 ± 6.6	<0.001	50.4 ± 6.2	50.0 ± 7.1	51.0 ± 5.8	50.0 ± 7.3	0.698
Mental Component Summary	53.1 ± 5.0	50.9 ± 8.3	0.036	50.9± 7.6	50.9 ± 8.7	51.1 ± 8.5	50.5 ± 8.8	0.970

Values are mean ± standard deviation or number. HEALTHY: healthy control; AC: attention control group; MICT: moderate-intensity continuous training group; HIIT: high-intensity interval training group; BMI: body mass index; SBP: systolic blood pressure; DBP: diastolic blood pressure; V̇O_2peak_: peak oxygen uptake. *p* < 0.05.

**Table 2:** Health-related Quality of Life outcomes before and after a 16-week intervention program in the EXERDIET-HTA group.

Variables	ALL (n = 253)	AC (n = 67)	High-Volume MICT (n = 61)	High-Volume HIIT (n = 60)	Low-VolumeHIIT (n = 65)	*p*_AC vs. ExT_	*p* Value Intergroups
Physical Functioning							
T_0_	88.9 ± 11.8	90.0 ± 10.3	88.5 ± 12.6	90.0 ± 10.8	87.3 ± 13.5	0.011	0.057
T_1_	93.3 ± 7.9 *^×^	91.4 ± 9.9	94.1 ± 6.9 *	94.4 ± 7.5 *	93.8 ± 6.6 *		
Role-Physical							
T_0_	91.8 ± 23.2	93.6 ± 20.5	89.1 ± 25.8	95.4 ± 18.1	90.2 ± 25.8	0.145	0.292
T_1_	93.6 ± 22.2	90.2 ± 28.6	95.0 ± 18.2	94.5 ± 17.8	94.5 ± 22.3		
Bodily Pain							
T_0_	78.8 ± 19.1	75.4 ± 19.8	79.2 ± 17.9	82.4 ± 16.4	77.8 ± 21.4	0.984	0.312
T_1_	81.4 ± 20.1	78.1 ± 23.5	85.5 ± 19.9	79.8 ± 19.2	82.2 ± 18.4		
General Health							
T_0_	64.1 ± 16.6	65.9 ± 18.3	64.4 ± 15.1	63.5 ± 14.7	62.4 ± 17.9	0.001	0.003
T_1_	71.0 ±17.1 *^×^	66.8 ± 21.3	70.6 ± 14.6 *	74.3 ± 15.5 *^×^	72.5 ± 15.5 *^×^		
Vitality							
T_0_	59.0 ± 14.9	58.5 ± 15.8	57.9 ± 15.1	61.8 ± 13.5	58.0 ± 15.2	0.101	0.043
T_1_	66.2 ± 13.8 *^×^	63.0 ± 15.9 *	66.3 ± 13.4 *	66.5 ± 13.5 *	69.1 ± 11.8 *		
Social Functioning							
T_0_	88.9 ± 17.0	89.2 ± 17.0	88.6 ± 16.7	90.0 ± 16.4	87.7 ± 18.0	0.951	0.769
T_1_	92.8 ± 14.6 *	93.0 ± 13.2	91.8 ± 19.6	92.5 ± 11.7	93.8 ± 13.2 *		
Role-Emotional							
T_0_	90.5 ± 26.7	93.2 ± 23.8	87.3 ± 29.0	93.3 ± 23.5	88.1 ± 30.2	0.100	0.157
T_1_	92.0 ± 23.5	89.8 ± 26.4	92.7 ± 21.9	92.1 ± 24.0	94.9 ± 18.4		
Mental Health							
T_0_	76.8 ± 14.1	76.1 ± 13.7	78.6 ± 13.3	76.6 ± 15.4	76.0 ± 14.2	0.287	0.509
T_1_	79.5 ± 12.9 *	77.1 ± 13.5	81.3 ± 12.7	78.8 ± 14.1	80.9 ± 11.3 *		
Physical Component Summary							
T_0_	50.6 ± 6.4	50.6 ± 6.1	50.3 ± 6.8	51.4 ± 5.5	50.0 ± 7.2	0.098	0.309
T_1_	52.3 ± 6.1	51.1 ± 7.4	52.9 ± 5.6*	53.0 ±5.0	52.6 ± 5.8 *		
Mental Component Summary							
T_0_	51.2 ± 8.3	51.3 ± 7.4	51.2 ± 8.8	51.6 ± 8.5	50.7 ± 8.7	0.329	0.396
T_1_	52.8 ± 7.3	52.0 ± 8.2	53.0 ± 8.1	52.5 ± 7.2	53.9 ± 5.3 *		

Values are mean ± SD. AC: attention control group; MICT: moderate-intensity continuous training group; HIIT: high-intensity interval training group; ExT: Exercise Training Groups; T_0_: pre intervention; T_1_: post-intervention. * *p-*value < 0.05 from T_0_; *^×^ *p-*value < 0.001 from T_0_.
